# Relationship of perinatal outcomes to the competence and quantity of contact with community health workers

**DOI:** 10.7189/jogh.15.04094

**Published:** 2025-02-28

**Authors:** Mark Tomlinson, Mary Jane Rotheram-Borus, Linnea Stansert Katzen, William Gertsch, Ingrid le Roux, Elaine Dippenaar, Karl le Roux

**Affiliations:** 1 Institute for Life Course Health Research, Department of Global Health, Faculty of Medicine and Health Sciences, Stellenbosch University, Tygerberg, South Africa; 2 School of Nursing and Midwifery, Queens University, Belfast, UK; 3 Department of Psychiatry and Biobehavioral Sciences, Semel Institute, University of CA, Los Angeles, California, USA; 4 Zithulele Training and Research Centre, Zithulele Hospital, Mqanduli District, Eastern Cape, South Africa; 5 Philani Maternal, Child Health and Nutrition Trust, Khayelitsha, Cape Town, South Africa; 6 Department of Family Medicine, Walter Sisulu University, Mthatha, South Africa; 7 Primary Health Care Directorate, Old Main Building, Groote Schuur Hospital, Cape Town, South Africa

## Abstract

**Background:**

The effectiveness of perinatal home visits by community health workers (CHWs) often diminishes when large regional or national programmes are implemented. To address this gap, we aimed to identify which CHW behaviours influence maternal and child outcomes.

**Methods:**

We randomised all government-funded CHWs at eight deeply rural clinics (n = 43) by clinic to usual care, which consisted of home visits (control group; four clinics, 23 CHWs, 392 mothers), or to home visiting, which included improved monitoring and supervision (intervention group; four clinics, 20 CHWs, 423 mothers). Since fewer than 7% of CHWs in the control group ever implemented home visits and no data was available on the frequency of visits, we focussed on the CHWs in the intervention group. We monitored the number and timing of home visits over time and documented it by paper and mobile phone records. Supervisors who conducted at-home observations of visits completed competency ratings on each CHW. We evaluated the associations between the competency of the CHW and the number and timing of CHWs’ visits with 13 maternal/child outcomes using multiple regression analyses.

**Results:**

Consistent home visits by CHWs reached the threshold at about 9–12 months, with the frequency reducing because of COVID-19. There were two significant outcomes (antiretroviral therapy adherence and securing the child grant) associated with the number of home visits in the intervention group, but insufficient to demonstrate efficacy. The CHW competency was unrelated to any maternal/child outcome. Moreover, CHWs visited 7% of mothers during the first two days of their infants’ lives, 26% during the first week, 57% within the first month, and 90% by the first three months of life.

**Conclusions:**

Current standards for training and monitoring of paraprofessional home visitors are highly unrealistic. Substantial and ongoing investments are needed for visits to occur consistently over time. However, hiring and selection criteria are likely as important as training and monitoring. CHW programmes must be embedded in organisational contexts that are well functioning and have management and support structures that are operational to ensure their success.

Keywords

There are about two million community health workers (CHWs) globally [1] who are being trained to provide support to professionals and, in particular, extend health care support into the community [2,3]. The World Health Organization (WHO) has advocated for a doubling of the number of CHWs to four million, given the dearth of health care professionals that is likely to persist until 2050 [4]. South Africa has 55 000 CHWs [5] who are a key component of the government's plans to ‘Re-engineer Primary Health Care’ [6-9]. In 2011, more than 33 000 of them were re-assigned from being based mostly at clinics to conducting home visits [10,11]. We aimed to examine the operational challenges of deploying these CHWs and the outcomes of having CHWs conduct perinatal home visits in deeply rural communities.

Evaluations of CHW impact have been mixed [12,13]. Several efficacy trials have found benefits associated with CHWs [12,14,15]. In a randomised controlled trial (RCT) in 24 peri-urban Cape Town neighbourhoods, our team found significant benefits of perinatal CHW home visiting that lasted for up to five years for mothers and three years for their children [16–19], but dissipated by eight years post-birth [20]. In rural settings, two trials with comparison communities (not RCTs) were conducted, again finding benefits, although more limited to mental health benefits [21–23].

We aimed to evaluate the impact of CHW home visiting when researchers have no role in CHW selection, but when training, monitoring, and supervision are systematically implemented by a non-governmental organisation. In a recent publication [20], 11 of 13 maternal and child outcomes favoured families where the CHW had received additional supervision and monitoring. However, predefined criteria for evaluating efficacy did not show significant impacts on a sufficient number of outcomes over 24 months [20]. Given the close monitoring of CHWs over time, we wanted to examine whether specific CHW behaviours associated with the benefits of home visiting can be identified.

Most studies of interventions to improve the quality of CHW visits have focussed on enhanced knowledge or skills, with multiple evaluations of training packages having been conducted. Literacy, social skills, experience of being a mother, and being 30–50 years old have been identified as the key characteristics of a CHW within the health system [24]. While a long list of supportive conditions has been identified, a significant gap remains between recognising what is needed and what happens in practice [24,25]. The complexity of implementation has recently been highlighted by the Sustainable Programme Incorporating Nutrition and Games (SPRING) trial, which attempted to improve child developmental outcomes by embedding home visits into existing lady health worker visits in Pakistan and India [26]. The lack of any positive outcome was ascribed, in part, to shortcomings in effective implementation.

Supervision and accountability are critical components of delivering high-quality care. Yet while a meta-analysis of 14 interventions attempted to assess the benefits of supervision of CHWs, it failed to do so due to the varying quality of the included studies [27]. A more recent study [28] found that supervision was unrelated to outcomes. Only case studies provided qualitative data on the key dimensions of relevant supervision [29].

In a real-world setting in which CHWs have been redeployed to provide home visits in rural Eastern Cape of South Africa, we examined the competence of CHWs, the number of visits conducted over time, and the association of these variables to 13 maternal and child outcomes over the first two years of life.

## METHODS

We conducted this study in the deeply rural King Sabata Dalindyebo Health Sub-district of the Oliver Reginald Tambo District, Eastern Cape of South Africa. The catchment area was served by eight primary care clinics that refer patients to Zithulele District Hospital, a state-run facility that serves about 130 000 people [2]. Only 27% of households have access to communal taps, with 48% relying on unsafe river water, while 93% of households receive a government grant [30]. Before randomisation, we documented the size of the clinics’ catchment areas, the density of housing in the catchment area, and the number of women initiating antenatal care over the previous year.

A consecutive series of mothers presenting as pregnant at four clinics (n = 450) were recruited and monitored by an independent team of assessors in pregnancy. They were followed up at three, six, 15-, and 24-month post-birth, with follow-up rates ranging between 76% and 86%.

Local women who lived in adjoining neighbourhoods were recruited and trained as data collectors. They were all isiXhosa-speaking women who also spoke English, typically had a 12th-grade education, and were selected based on their good social skills, the ability to engage peers, and the responsibility and ability to complete interviews on a digital platform. They were assigned to clinics during the recruitment period and conducted all assessments in the participants' homes or outdoors if there was no privacy in the home. They were blinded to the intervention condition, but may have realised its status over time based on mothers’ answers to assessment questions.

There were 23 CHWs at the four intervention group clinics [1]. They had a mean age of 41 (SD = 9.3). Almost two-thirds had less than a high school education, with eight (35%) graduating from high school. More than half (56%) were single, two (8.7%) were widowed, and eight (35%) were married/living with a partner. There were 20 (87%) CHWs with children at home and five (22%) had grandchildren. On average, they had 11.6 years (SD = 7.0) of experience in their job and carried a caseload of 35 households (SD = 12.1).

Mothers reported visits by a CHW at each assessment, while CHWs recorded their visits with a GPS finder and a paper-based report, as well as having a mobile phone which allowed tracking of the visit and communication with supervisors, similar to recommendations regarding optimal practice [31]. The total number of visits was calculated as the sum of the number of antenatal, first 48 hours, 48 hours to one week, one week to one month, one month to three months, and three months to 6-month visits.

Supervisors rated the competency of CHWs, with 12 domains of functioning rated for each CHW by at least two supervisors who observed the day-to-day implementation of home visiting. Each domain was rated on a 1–4 scale, including social skills, problem-solving, social connections, general performance, consistency of follow-up, content relevance, case-seeking, adhering to administrative procedures, identifying serious cases, and completing folders. The total competence score was the sum of ratings across 12 domains, where each domain was given a score of poor (1), fair (2), or good (3).

### Measures

The following measures were gathered from each mother and used in the evaluation of client outcomes over 24 months [20]:

− Alcohol use during pregnancy – mothers were asked if they ever used alcohol after discovering they were pregnant (1) or not (0) before birth.
− Depressive symptoms – reported at each assessment using the Edinburgh Postnatal Depression Scale [31]. Mothers with a score ≥13 were considered to have a depressed mood (1) at any assessment over 24 months or not (0).
− Antenatal adherence to four healthcare visits (1) or not (0).
− Adherence to tasks to prevent mother-child transmission by mothers living with HIV (MLH) – based on a summary count of the following tasks the MLH completed, as recorded on her ‘Road to Health’ card (RTHC): exclusively breastfed for six months; giving nevirapine at birth; giving Bactrim for six weeks; testing the child for HIV before three months of age; and going to the clinic to receive the results of the baby’s HIV test.
− Adherence to antiretroviral therapy (ART) for MLH – the summed total of assessments at which MLH self-reported adhering to ART as ‘very good’ or ‘excellent’.
− Breastfeeding for at least six months – self-reported by mothers and calculated as yes (0) or not (1) at both the three and six-month assessments.
− Having a low birth weight infant (*i.e.* less than 2500 grams) (0) or not (1) based on the child’s RTHC.
− Having a stunted (−2.9 standard deviation (SD) on height-per-age Z-score) at any assessment over 24 months.
− Having a malnourished child (−2 SD on weight-per-age Z-score) at any assessment over 24 months.
− Securing the child support grant by six months (0) or not (1).
− Immunisations – classed as up to date at each assessment (1) or not (0) based on the child’s RTHC.
− Child hospitalisations – recorded as occurring (1) or not (0) over 24 months based on the child’s RTHC.
− Developmental milestones – based on a count of the WHO-stated developmental milestones [26,32] at six, 15, and 24 months.

### Data analysis

We had two primary research questions:

− Does the number of visits influence maternal/child health outcomes over two years?
− Does the total competence score of the CHW influence maternal/child health outcomes?

We evaluated these questions independently using linear regression for continuous outcomes and logistic regression for binary outcomes. Even though there was a clustering of participants into clinics, there were only four clinics, so we decided to omit the random effect and performed a sensitivity analysis which included the clinic-level random effect. We performed power and sample size calculations for the main analysis comparing standard of care and accountable care [20]. We assessed significance using two-sided tests at an alpha (α) level of 0.05. Since the 13 outcomes were correlated, we performed a *post-hoc* analysis of the number of significant results we observed in our analysis. We applied the procedure of Harwood and colleagues [33] with an estimated correlation of 0.10 and found that three significant outcomes are required to declare a significant effect for each of the independent variables.

## RESULTS

Mothers were on average 25.3 (SD = 6.5) years old and 108 (24%) were married or had a live-in partner. On average, there were 3.3 adults (SD = 1.7) and three children (SD = 2.1) in their household. Only 180 (40%) reported consistently having a secure food source. Almost all income was from grants, with 378 (84%) receiving government grants and 392 (87%) receiving a child grant. Six (1.3%) had attempted suicide, 17 (3.7%) reported a depressed mood, and 18 (4%) used alcohol after discovering they were pregnant. There were 153 (34%) mothers who were MLH and more than 99% had received ART to treat their disease. One-fifth (n = 99, 22%) had experienced interpersonal violence in their lifetime, with 68 (15%) experiencing it recently.

**Figure 1:**
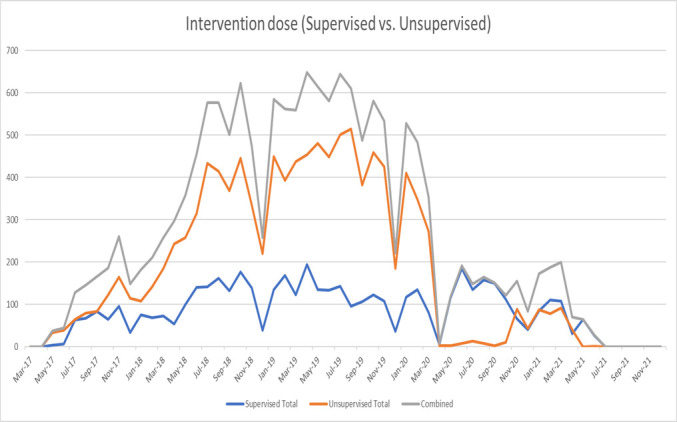
The frequency of visits over time by the CHWs from four clinics in the rural area of Zithulele, including supervised, unsupervised, and total visits. CHW – community health worker.

It took almost a year for the activity level of CHWs to be at its highest (**Figure 1**). There was a fall in visits annually during the Christmas break (a typical six-week break nationally). The frequency of home visits rapidly declined after March 2020, when COVID-19 lockdowns were implemented in South Africa; it was slow to increase when they were lifted, and the study period ended before there had been a resumption to the activity level which had existed pre-lockdown.

Among new mothers, 32 (7%) were visited in the first two days of returning from the hospital, 117 (26%) were visited in the first week, 257 (57%) were visited within their first month, 360 (80%) were visited within the first three months, and 405 (90%) were visited in the first six months of life.

We obtained two significant outcomes for the number of visits: receipt of a child support grant and adherence to child HIV prevention measures (**Table 1**). However, our simulations indicated that we needed to find three significant outcomes to be considered significantly different, which is why we can conclude that number of visits was unrelated to maternal-child outcomes. There was also no significant effect on maternal outcomes based on supervisors’ ratings of CHW competence (**Table 2**). Similar to the number of visits, CHW competence did not affect outcomes.

**Table 1:** Results of the regression analysis of the total number of visits made by CHWs on maternal outcomes over 24 months

	*β* (95% CI)	*P*-value
Binary outcomes*		
Breastfeeding at six months	0.99 (−0.94, 1.04)	0.682
Low birth weight	0.92 (0.85, 1.01)	0.076
Alcohol during pregnancy	1.02 (0.89, 1.16)	0.799
Depressed	0.99 (0.90, 1.09)	0.875
WAZ<−2.9	1.08 (0.93, 1.27)	0.320
HAZ<−2.0	0.98 (0.92, 1.05)	0.652
Child support grant for six months	1.10 (1.01, 1.20)	0.035
Up-to-date immunisations	1.06 (0.98, 1.16)	0.147
No hospitalisations	1.00(0.95, 1.05)	0.978
Went to ≥4 antenatal doctor appointments	0.97 (0.91, 1.04)	0.437
**Continuous outcomes**		
Adherence to child HIV preventive measures (HIV+ mothers)	0.04 (0.00, 0.08)	0.047
Adherence to ART at all time points (HIV+ mothers)	−0.02 (−0.11, 0.07)	0.665
Number of developmental milestones	−0.03 (−0.07, 0.02)	0.209

ART – antiretroviral therapy, CI – confidence interval, CHW – community health worker, WAZ – weight-per-age Z-score, MLH – mothers living with HIV, HAZ – height-per-age Z-score

*The estimated coefficient is on the odds ratio scale.

**Table 2:** Results of the regression analysis of the supervisors’ summed scores on the competence of CHWs on maternal outcomes over 24 months

	*β* (95% CI)	*P*-value
Binary outcomes*		
Breastfeeding at six months	0.97 (0.94, 1.02)	0.275
Low birth weight	1.02 (0.96, 1.08)	0.586
Alcohol during pregnancy	1.02 (0.92, 1.12)	0.768
Depressed	1.07 (0.99, 1.15)	0.105
WAZ<−2.9	1.02 (0.89, 1.16)	0.822
HAZ<−2.0	1.02 (0.97, 1.07)	0.482
Child support grant for six months	1.02 (0.96, 1.08)	0.494
Up-to-date immunisations	1.03 (0.97, 1.09)	0.350
No hospitalisations	1.02 (0.98, 1.06)	0.260
Went to ≥4 antenatal doctor appointments	1.01 (0.95, 1.06)	0.817
**Continuous outcomes**		
Adherence to child HIV preventive measures (MLH)	0.01 (−0.03, 0.04)	0.651
Adherence to ART at all time points (MLH)	−0.05 (−0.14, 0.04)	0.319
Number of developmental milestones	0.00 (−0.03, 0.03)	0.998

ART – antiretroviral therapy, CI – confidence interval, CHW – community health worker, WAZ – weight-per-age Z-score, MLH – mothers living with HIV, HAZ – height-per-age Z-score

*The estimated coefficient is on the odds ratio scale.

## DISCUSSION

Health systems worldwide are addressing the shortage of professional providers by expanding the use of trained paraprofessionals, particularly CHWs. Currently, over two million CHWs are active in the field, many providing perinatal home visits. While several studies have demonstrated the potential efficacy of CHW programmes [34,35], their effectiveness has rarely been proven at scale [32,36]. These challenges have been observed in both high-income and low- and middle-income countries. A recent RCT on this topic was published, but did not show sufficient efficacy to support broader implementation [20].

Our study highlights the significant challenges of deploying CHWs for perinatal home visits in deep rural areas. Despite perinatal home visits being part of the job description, the CHWs in the control group conducted almost no visits, even though they all received an additional month of training. In the intervention group, it took approximately nine months for CHWs to reach the expected frequency of 4–6 home visits per day. Progress was slow despite routine monitoring, weekly reviews of visit logs, and supervisors attending about one-third of the visits. Only after nine months did CHWs consistently reach their assigned goals. Given that CHW training globally typically lasts just one week and supervisor site visits are uncommon, these findings suggest a significant underestimation of the resources required to develop a strong global CHW workforce capable of delivering high-quality services to families.

Even more concerning are the findings on the relationship between monitoring, supervision frequency, and the perceived CHW competency with programme outcomes. Only two significant outcomes were related to visit frequency – adherence to ART and securing the child grant. This limited number of significant findings is insufficient to demonstrate a relationship between overall outcomes and the intensity of perinatal visits. Additionally, supervisors’ ratings of CHWs’ quality of care showed no association with maternal and child outcomes.

There is insufficient data on the timing of visits in broad-based behavioural interventions like ours to determine whether it influences outcomes. However, strong evidence suggests that timing plays a crucial role in areas such as reducing neonatal mortality. In our study, likely influenced by travel distance [37], only 7% of visits in the intervention group occurred within the first two days, increasing to 26% within the first week and over 75% within the first month. While these rates are likely higher than typical, there is still significant room for improvement. Given these findings, it is essential to examine why the impact remains so limited.

Several factors may explain why it took CHWs so long to reach the targeted number of home visits. First, many CHWs had been working for years in clinic-based roles before being assigned to perinatal home visits. Transitioning from clinic work to home visits may have been challenging, both for the CHWs themselves and for their supervisors and clinic nurses, who were accustomed to having paraprofessional support within the clinic setting. Mandating job reassignments in health systems with diverse personnel roles is unlikely to be effective, and this challenge was particularly evident in this deeply rural setting.

Second, the research and implementation teams had no control over CHW selection. Recruitment had taken place earlier and was typically based on referrals from local tribal chiefs or leaders. In contrast, previous successful interventions using the Philani model involved a structured recruitment process managed by Philani. This process ensured that recruited CHWs were positive role models (*i.e.* had good social skills and families whose children were doing well) and underwent rigorous screening by multiple stakeholders both before and during training. In the Philani model, becoming a home visitor required assessments by supervisors, other CHWs, and direct observations during home visits. However, such selection, interviewing, and screening criteria are not standard in most CHW hiring processes, including in South Africa. Even with a training period four times longer than typical programmes (*i.e.* one month), many CHWs struggled to perform the duties associated with home visiting.

Finally, several methodological issues may have contributed to the lack of observed differences. These analyses lacked a true contrast group, as CHWs in the control group had no records of any home visits and conducted few visits. It is possible that CHW skill levels were either universally high or low, decreasing the probability of significant effects. Additionally, the existing clinic-based care system may have been robust enough to decrease the need for CHWs’ home visits. When assessing the overall effectiveness of this RCT, the anticipated risks among mothers were lower than expected [20]. Alcohol use and depression rates were low, while breastfeeding rates and the rate of obtaining the child grant were substantially higher [20]. Given these conditions, the benefits associated with the home visits were likely reduced. Furthermore, at the time of our study, antenatal visit attendance was strong, and families had access to Zithulele Hospital, which was recognised as a model for high-quality rural health care [38].

## CONCLUSIONS

Our findings add to the already complex understanding of CHW effectiveness in rural settings, particularly where recruitment, training, and supportive supervision are limited. For CHW programmes to succeed, they must be integrated into well-functioning organisational structures with effective management and support systems [39]. Based on our results, it may not be an overstatement to suggest that in this setting, where management and accountability mechanisms appear to be absent, CHW home visiting programmes may not be worth the investment. At the very least, serious consideration should be given to alternative models of care delivery.

## References

[1] Shah R Camarena A Park C Martin A Clark M Atkins M Healthcare-Based Interventions to Improve Parenting Outcomes in LMICs: A Systematic Review and Meta-Analysis. Matern Child Health J. 2022;26:1217–30. 10.1007/s10995-022-03445-y 35579803

[2] Zulu JM Perry HB Community health workers at the dawn of a new era. Health Res Policy Syst. 2021;19:130. 10.1186/s12961-021-00761-7 34641904 PMC8507089

[3] Chowdhary N Sikander S Atif N Singh N Ahmad I Fuhr DC The content and delivery of psychological interventions for perinatal depression by non-specialist health workers in low and middle income countries: a systematic review. Best Pract Res Clin Obstet Gynaecol. 2014;28:113–33. 10.1016/j.bpobgyn.2013.08.013 24054170 PMC3893480

[4] World Health Organization. Community Health Worker Programmes in the WHO African Region: Evidence and Options — Policy brief. Geneva, Switzerland: World Health Organization; 2017. Available: https://www.afro.who.int/sites/default/files/2017-07/Community%20Health%20Worker%20Policy%20Brief%20-%20English_0.pdf. Accessed: 8 February 2025.

[5] Health Systems Trust. District Health Barometer: District Health Profiles. Durban, South Africa: Health Systems Trust; 2018. Available: https://www.hst.org.za/publications/District%20Health%20Barometers/Distric%20Health%20Barometer-District%20Health%20Profiles%2020172018.pdf. Accessed: 8 February 2025.

[6] Austin-Evelyn K Rabkin M Macheka T Mutiti A Mwansa-Kambafwile J Dlamini T Community health worker perspectives on a new primary health care initiative in the Eastern Cape of South Africa. PLoS One. 2017;12:e0173863. 10.1371/journal.pone.0173863 28301609 PMC5354377

[7] le Roux K le Roux I Mbewu N Davis E The Role of Community Health Workers in the Re-Engineering of Primary Health Care in Rural Eastern Cape. S Afr Fam Pract (2004). 2015;57:116–120. 10.1080/20786190.2014.977063 26279948 PMC4532349

[8] Siruma A Hornby D Srinivas S An assessment of maternal health issues in two villages in the Eastern Cape province of South Africa. Int J Environ Res Public Health. 2014;11:9871–84. 10.3390/ijerph110909871 25247428 PMC4199055

[9] Swartz A Colvin CJ ‘It’s in our veins’: Caring Natures and Material Motivations of Community Health Workers in Contexts of Economic Marginalisation. Crit Public Health. 2015;25:139–52. 10.1080/09581596.2014.941281

[10] Pillay Y, Barron P. The implementation of PHC re-engineering in South Africa. 2010. Available: https://phasa.org.za/index.php/resources/blogs-articles/pdf-listing/download-file?path=Pillay-The-implementation-of-PHC.pdf. Accessed: 19 February 2025.

[11] Schneider H, Daviaud E, Besada D, Rohde S, Sanders D. Ward-based primary health care outreach teams in South Africa: developments, challenges and future direction directions. In: Rispel LC, Padarath A, editors. South African Health Review 2018. Durban, South Africa: Health Systems Trust; 2018. p. 59–66.

[12] Perry HB Zulliger R Rogers MM Community health workers in low-, middle-, and high-income countries: an overview of their history, recent evolution, and current effectiveness. Annu Rev Public Health. 2014;35:399–421. 10.1146/annurev-publhealth-032013-182354 24387091

[13] Wilford A Phakathi S Haskins L Jama NA Mntambo N Horwood C Exploring the care provided to mothers and children by community health workers in South Africa: missed opportunities to provide comprehensive care. BMC Public Health. 2018;18:171. 10.1186/s12889-018-5056-y 29361926 PMC5781263

[14] Kok M Crigler L Musoke D Ballard M Hodgins S Perry HB Community health workers at the dawn of a new era: 10. Programme performance and its assessment. Health Res Policy Syst. 2021;19:108. 10.1186/s12961-021-00758-2 34641901 PMC8506096

[15] Scott K Beckham SW Gross M Pariyo G Rao KD Cometto G What do we know about community-based health worker programs? A systematic review of existing reviews on community health workers. Hum Resour Health. 2018;16:39. 10.1186/s12960-018-0304-x 30115074 PMC6097220

[16] Tomlinson M Rotheram-Borus MJ le Roux IM Youssef M Nelson SH Scheffler A Thirty-Six-Month Outcomes of a Generalist Paraprofessional Perinatal Home Visiting Intervention in South Africa on Maternal Health and Child Health and Development. Prev Sci. 2016;17:937–48. 10.1007/s11121-016-0676-x 27438294 PMC5111552

[17] Rotheram-Borus MJ Richter LM van Heerden A van Rooyen H Tomlinson M Harwood JM A cluster randomized controlled trial evaluating the efficacy of peer mentors to support South African women living with HIV and their infants. PLoS One. 2014;9:e84867. 10.1371/journal.pone.0084867 24465444 PMC3898948

[18] Tomlinson M Rotheram-Borus MJ Harwood J le Roux IM O’Connor M Worthman C Community health workers can improve child growth of antenatally-depressed, South African mothers: A cluster randomized controlled trial. BMC Psychiatry. 2015;15:225. 10.1186/s12888-015-0606-7 26400691 PMC4581418

[19] le Roux IM Tomlinson M Harwood JM O’Connor MJ Worthman CM Mbewu N Outcomes of home visits for pregnant mothers and their infants: a cluster randomised controlled trial. AIDS. 2013;27:1461–71. 10.1097/QAD.0b013e3283601b53 23435303 PMC3904359

[20] Rotheram-Borus MJ le Roux KW Norwood P Stansert Katzen L Snyman A le Roux I The effect of supervision on community health workers’ effectiveness with households in rural South Africa: A cluster randomized controlled trial. PLoS Med. 2023;20:e1004170. 10.1371/journal.pmed.1004170 36862754 PMC9980736

[21] le Roux IM le Roux K Mbeutu K Comulada WS Desmond KA Rotheram-Borus MJ A randomized controlled trial of home visits by neighborhood mentor mothers to improve children’s nutrition in South Africa. Vulnerable Child Youth Stud. 2011;6:91–102. 10.1080/17450128.2011.564224 22299019 PMC3262232

[22] le Roux KW Almirol E Rezvan PH le Roux IM Mbewu N Dippenaar E Community health workers impact on maternal and child health outcomes in rural South Africa - a non-randomized two-group comparison study. BMC Public Health. 2020;20:1404. 10.1186/s12889-020-09468-w 32943043 PMC7496216

[23] Stansert Katzen L le Roux KW Almirol E Hayati Rezvan P le Roux IM Mbewu N Community health worker home visiting in deeply rural South Africa: 12-month outcomes. Glob Public Health. 2021;16:1757–70. 10.1080/17441692.2020.1833960 33091320

[24] Surjaningrum ER Minas H Jorm AF Kakuma R The feasibility of a role for community health workers in integrated mental health care for perinatal depression: a qualitative study from Surabaya, Indonesia. Int J Ment Health Syst. 2018;12:27. 10.1186/s13033-018-0208-0 29881450 PMC5984432

[25] Olal E Umar N Anyanti J Hill Z Marchant T How valid are women’s reports of the antenatal health services they receive from Community Health Workers in Gombe State north-eastern Nigeria? BMC Pregnancy Childbirth. 2022;22:898. 10.1186/s12884-022-05220-x 36463102 PMC9719641

[26] Kirkwood BR Sikander S Roy R Soremekun S Bhopal SS Avan B Effect of the SPRING home visits intervention on early child development and growth in rural India and Pakistan: parallel cluster randomised controlled trials. Front Nutr. 2023;10:1155763. 10.3389/fnut.2023.1155763 37404861 PMC10315474

[27] Ballard M Montgomery P Systematic review of interventions for improving the performance of community health workers in low-income and middle-income countries. BMJ Open. 2017;7:e014216. 10.1136/bmjopen-2016-014216 29074507 PMC5665298

[28] Francetic I Tediosi F Salari P de Savigny D Going operational with health systems governance: supervision and incentives to health workers for increased quality of care in Tanzania. Health Policy Plan. 2019;34:ii77–92. 10.1093/heapol/czz104 31723971

[29] Westgate C Musoke D Crigler L Perry HB Community health workers at the dawn of a new era: 7. Recent advances in supervision. Health Res Policy Syst. 2021;19:114. 10.1186/s12961-021-00754-6 34641909 PMC8507092

[30] Le Roux K Akin-Olugbade O Katzen LS Laurenzi C Mercer N Tomlinson M Immunisation coverage in the rural Eastern Cape — are we getting the basics of primary care right? Results from a longitudinal prospective cohort study. S Afr Med J. 2016;107:52–5. 10.7196/SAMJ.2017.v107i1.11242 28112092 PMC5659717

[31] Braun R Catalani C Wimbush J Israelski D Community health workers and mobile technology: a systematic review of the literature. PLoS One. 2013;8:e65772. 10.1371/journal.pone.0065772 23776544 PMC3680423

[32] Tomlinson M Rahman A Sanders D Maselko J Rotheram-Borus MJ Leveraging paraprofessionals and family strengths to improve coverage and penetration of nutrition and early child development services. Ann N Y Acad Sci. 2014;1308:162–71. 10.1111/nyas.12269 24117669 PMC4005291

[33] Harwood JM Weiss RE Comulada WS Beyond the Primary Endpoint Paradigm: A Test of Intervention Effect in HIV Behavioral Intervention Trials with Numerous Correlated Outcomes. Prev Sci. 2017;18:526–33. 10.1007/s11121-017-0788-y 28434056 PMC5627604

[34] Cooper PJ Tomlinson M Swartz L Landman M Molteno C Stein A Improving quality of mother-infant relationship and infant attachment in socioeconomically deprived community in South Africa: randomised controlled trial. BMJ. 2009;338:b974. 10.1136/bmj.b974 19366752 PMC2669116

[35] Tomlinson M Doherty T Ijumba P Jackson D Lawn J Persson LA Goodstart: a cluster randomised effectiveness trial of an integrated, community-based package for maternal and newborn care, with prevention of mother-to-child transmission of HIV in a South African township. Trop Med Int Health. 2014;19:256–66. 10.1111/tmi.12257 24433230

[36] Tomlinson M Rotheram-Borus MJ Swartz L Tsai AC Scaling up mHealth: where is the evidence? PLoS Med. 2013;10:e1001382. 10.1371/journal.pmed.1001382 23424286 PMC3570540

[37] Baqui AH Ahmed S El Arifeen S Darmstadt GL Rosecrans AM Mannan I Effect of timing of first postnatal care home visit on neonatal mortality in Bangladesh: a observational cohort study. BMJ. 2009;339:b2826. 10.1136/bmj.b2826 19684100 PMC2727579

[38] Baleta A Rural hospital beats the odds in South Africa. Lancet. 2009;374:771–2. 10.1016/S0140-6736(09)61577-4 19743534

[39] Tomlinson M Hunt X Rotheram-Borus MJ Diffusing and scaling evidence-based interventions: eight lessons for early child development from the implementation of perinatal home visiting in South Africa. Ann N Y Acad Sci. 2018;1419:218–29. 10.1111/nyas.13650 29791741

